# Long-term oxytocin administration improves social behaviors in a girl with autistic disorder

**DOI:** 10.1186/1471-244X-12-110

**Published:** 2012-08-13

**Authors:** Hirotaka Kosaka, Toshio Munesue, Makoto Ishitobi, Mizuki Asano, Masao Omori, Makoto Sato, Akemi Tomoda, Yuji Wada

**Affiliations:** 1 Research Center for Child Mental Development, University of Fukui, Eiheiji, Fukui, 910-1193, Japan; 2 Department of Neuropsychiatry, Faculty of Medical Sciences, University of Fukui, Eiheiji, Fukui, 910-1193, Japan; 3 Research Center for Child Mental Development, Kanazawa University, Kanazawa, Ishikawa, 920-8641, Japan; 4 Faculty of Nursing and Social Welfare Sciences, Fukui Prefectural University, Eiheiji, Fukui, 910-1195, Japan; 5 Division of Cell Biology and Neuroscience, Department of Morphological and Physiological Sciences, Faculty of Medical Sciences, University of Fukui, Eiheiji, Fukui, 910-1193, Japan

**Keywords:** Autism spectrum disorders (ASDs), Oxytocin, Social impairments

## Abstract

**Background:**

Patients with autism spectrum disorders (ASDs) exhibit core autistic symptoms including social impairments from early childhood and mostly show secondary disabilities such as irritability and aggressive behavior based on core symptoms. However, there are still no radical treatments of social impairments in these patients. Oxytocin has been reported to play important roles in multiple social behaviors dependent on social recognition, and has been expected as one of the effective treatments of social impairments of patients with ASDs.

**Case presentation:**

We present a case of a 16-year-old girl with autistic disorder who treated by long-term administration of oxytocin nasal spray. Her autistic symptoms were successfully treated by two month administration; the girl’s social interactions and social communication began to improve without adverse effects. Her irritability and aggressive behavior also improved dramatically with marked decreases in aberrant behavior checklist scores from 69 to 7.

**Conclusion:**

This case is the first to illustrate long-term administration of oxytocin nasal spray in the targeted treatment of social impairments in a female with autistic disorder. This case suggests that long-term nasal oxytocin spray is promising and well-tolerated for treatment of social impairments of patients with ASDs.

## Background

Autism spectrum disorders (ASDs) are a neuropsychiatric condition characterized by the disruption of the development of social intelligence [1], and marked impairments in social interactions and social communication, alongside the presence of repetitive behaviors and restricted interests. Social impairments including the inability to interpret others’ behavior in terms of mental states and to interact both in complex social groups and in close relationships are the main symptoms of patients with ASDs [1] and also induce secondary disabilities such as irritability, aggressive behavior, and/or depressed mood. Although many clinicians and psychologists attempt a host of psychotropic medications and psychoeducational treatments, there are still no radical treatments of social impairments of patients with ASDs.

Recently, many researchers have been paying attention to oxytocin, a neuropeptide that is secreted from the posterior pituitary, as a promising candidate treatment of social impairments of ASD patients. Oxytocin plays important roles in social memory and behaviors dependent on social recognition, such as pair bonding, mate guarding, and parental care in rodents [2]. Moreover, in non-ASD subjects, several studies suggest that nasal administration of a single dose of oxytocin improves the feeling of trust, ability to infer the mental state of others, and time of gazing toward the eye region, which are frequent deficits of ASD patients [3,4]. In addition, in ASD patients whose plasma oxytocin level is reported to be low [5], nasal administration of a single dose of oxytocin improves emotional recognition, and the patients exhibit more appropriate social behavior and affect [6,7]. Considering these results obtained by even a single dose of oxytocin, oxytocin is expected as one of the effective treatments of social impairments of patients with ASDs [6,7].

However, oxytocin is off-label for ASD treatment, and many questions still remain about the wider physiological effects of oxytocin and its safety on humans [8]. Regarding the long-term use of oxytocin, our recent case report has been the only one available; among the findings of that study, an autistic boy showed improvement of some social interactions such as mind or emotion reading, social memory, and positive communication after one year of oxytocin administration [9]. However, there have been no reports of long-term oxytocin treatment of female patients with ASDs; probably, clinicians do not administer oxytocin to female patients because they are concerned about female-specific adverse effects of oxytocin treatment such as milk ejection and uterine contractions.

We here report the case of a high-functioning female patient with autistic disorder, in whom oxytocin showed favorable effects on aberrant social behaviors without any adverse effects.

## Case presentation

A 16-year-old girl with her mother visited our department presenting with incomprehensibility of others’ mind and aggressive behaviors including self-injury. She showed a marked impairment in social interactions (i.e., having few friends, lack of empathy, poor understanding of others’ mind). She also showed echolalia in her childhood, and she was obsessed with playing video games and showed a strong interest in wearing flashy clothes. She had mood fluctuations and often upset other people with her temper tantrums. She had no history of neurological or psychiatric diseases, except for autistic symptoms, or maltreatment, and her brain magnetic resonance imaging (MRI) showed no apparent abnormal findings.

The author H.K. diagnosed her as having autistic disorder on the basis of the classifications in the Diagnostic and Statistical Manual of Mental Disorders, Fourth Edition, Text Revision (DSM-IV-TR; American Psychiatric Association, 2000) and standardized criteria using the Diagnostic Interview for Social and Communication Disorders (DISCO) [10]. Her full-scale intelligence quotient was 92, as determined using the Wechsler adult intelligence scale-third edition. Her aberrant behavior checklist (ABC) [11] completed by her mother at the initial visit showed a very high score of 69 for items such as hyperirritability, strong aggression, poor empathy, and bizarre behaviors. For several months, she received behavioral psychoeducational treatment as well as adequate trials of medications including risperidone and antianxiety agents; but these were ineffective and the medications caused extrapyramidal symptoms and drowsiness.

Then, her mother learned from the internet about the efficacy of nasal oxytocin spray for autism, and she decided to try it on her at an intranasal dose of 8 IU of oxytocin per day (Syntocinon spray, Novartis; one puff each in the morning and evening) for personal use. Her mother decided to use a smaller amount of intranasal oxytocin than that indicated in previous reports [3,4,6,7]. Using some checklists and by physical examinations, we monitored the patient’s behavioral symptoms and verified the mother's impressions and observations at every visit. One month after starting nasal oxytocin spray administration, the girl’s social behaviors began to improve. The duration in which she closeted herself in her room became short. She greeted other people and made small talk with them, and she also showed empathy for others’ sickness and worries. She became able to express gratitude to her family for their support. She became able to carefully listen to her family’s conversation, and showed attenuated expressions of rebellion to the family’s words of caution. Even when she lost her temper, she calmed down immediately. A teacher who taught her about culture and who did not know about her treatment noted decreases in the numbers of episodes of irritability and self-injury, and was surprised at the increases in the frequencies of daily conversations and happy facial expressions in the presence of other people. Her score in ABC completed by her mother markedly decreased from 69 to 15, two months after starting oxytocin treatment. Marked decreases were found in her subscale scores for the (I) Irritability and Agitation item (from 28 to 12) and (IV) Hyperactivity and Noncompliance item (from 20 to 2). Her score of the Clinical Global Impression (CGI) - Severity scale also decreased from 6 (“severely ill”) to 3 (“mildly ill”); her CGI-Improvement score was 1, which indicates "Very much improved". Even after six months of administration, her improved social behaviors continued with 7 of ABC scores completed by her mother. There were no obvious adverse effects of oxytocin, including anaphylactic reaction, nausea, or vomiting, as described in the package insert. Her menstrual cycle remained regular and milk ejection was not observed. No marked changes were observed in her sex hormone levels. A complete blood count test, renal and liver function tests, and brain MRI confirmed the absence of abnormal findings.

This is the first case report of a female patient with autistic disorder whose aberrant social behaviors and related autistic symptoms were improved by long-term nasal oxytocin administration. Although there was a study including two females with ASDs receiving a single dose of 24 IU oxytocin during a social game [7], there are no reports of long-term nasal oxytocin administration to female subjects with ASDs. The observed effects in the present case are in accordance with our previous report of an autistic boy receiving long-term oxytocin administration [9]. There is a possibility that her symptoms were improved by other confounding factors. However, she has not received any other medication since the administration of oxytocin nasal spray, and her symptoms had not been improved by psychoeducational treatment, which she received for several months before the administration of oxytocin. Although ABC and CGI used to assess this patient are subjective evaluation scales that may be influenced by the rater's expectations, for this patient, all the family members observed that her symptoms began to improve gradually and the improvements continued for more than six months. Moreover, other people associated with her such as her teacher also made similar observations.

Regarding safety issues, no adverse effects related to long-term oxytocin treatment were observed in the present case. This is partly because the expression level of oxytocin receptors in nonpregnant myometrium is markedly lower than that during the period of delivery [12], and the oxytocin dose of the present case is 8 IU per day, which is much lower than that (single dose of 24 IU oxytocin) in previous reports [3,4,6,7].

The amygdala is part of the "social brain" and is proposed to be one of the several neural regions that are abnormal in autism [1]. The amygdala theory of autism, which involves deficits in “social intelligence”, is supported by many researchers [1]. Regarding the association between the amygdala and the oxytocin receptor, the amygdala has abundant oxytocin receptors. A functional MRI study showed that a single dose of oxytocin attenuated amygdala responses to emotional faces [13]. It is also suggested that the rs2254298A allele of the oxytocin receptor, which is associated with ASDs, was significantly associated with larger bilateral amygdala volume [14]. Rosenfeld *et al.*[15] proposed a model comprising abnormalities in oxytocinergic and dopaminergic signalings in the amygdala that result in impaired emotional salience processing with consequent social cognitive deficits. Considering these findings, exogenous oxytocin administration may modulate these signalings in patients with social impairments, via the amygdala. Research on whether long-term administration of oxytocin leads to a decrease in expression levels of oxytocin receptors is also needed [8].

## Conclusions

This is the first case report of a female patient with autistic disorder whose social impairments and secondary disabilities were improved by long-term nasal oxytocin administration without any adverse effects. One of the limitations of this study is the potential problem in symptom severity assessment using subjective scales. In the near future, we will report our ongoing clinical research on the long-term administration of oxytocin in randomized, double-blind, placebo-controlled trials in both male and female youths with ASDs, to clarify the potential therapeutic role and safety of continuous nasal oxytocin administration. Although we should be careful about long-term oxytocin administration, this case suggests that long-term oxytocin administration is a potential safe and well-tolerated treatment for improving social impairments even in female patients with ASDs.

### Consent

Written informed consent was obtained from the patient for publication of this Case report.

## Competing interests

Authors have no competing interests to declare that are relevant to the content of this submission.

## Authors’ contributions

HK and MI reviewed the literature and wrote the report. MA, MO, MS, AT and YW took part in the scientific discussion and contributed to writing the case presentation. TM conceptualized and supervised the procedure. All authors read and approved the final manuscript.

## Pre-publication history

The pre-publication history for this paper can be accessed here:

http://www.biomedcentral.com/1471-244X/12/110/prepub
